# Supplementary material for: Causal Relationship between Aging and Anorexia Nervosa: A White-Matter-Microstructure-Mediated Mendelian Randomization Analysis
Source: Biomedicines. 2024 Aug 16;12(8):1874. doi: 10.3390/biomedicines12081874 (PMC11351342; doi:10.3390/biomedicines12081874)
Supplement: Supplementary file 1 [file biomedicines-12-01874-s001.zip › Supplementary Figure/Figure S8/Leaveoneout plot of MR analysis for TL to ubm-b-1938.pdf]

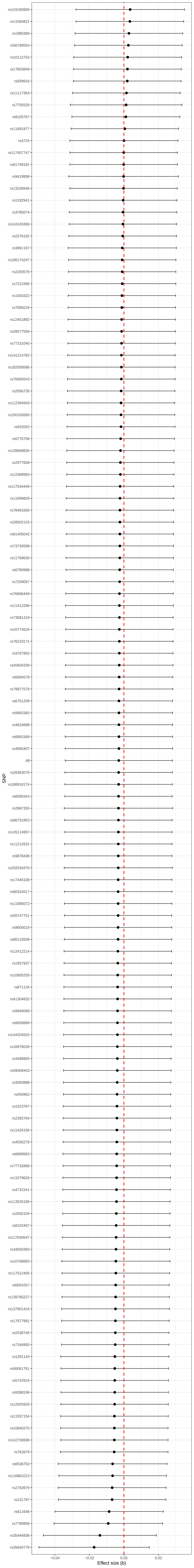
rs143190905
rs11584821
rs1985369
rs56799554
rs10112752
rs17803849
rs939916
rs11117354
rs7705526
rs8105767
rs11991877
rs4724
rs117407747
rs61748181
rs9419958
rs13230646
rs1332941
rs3785074
rs142426306
rs2276182
rs3891167
rs185174247
rs2293579
rs7221585
rs1003322
rs7099229
rs12451892
rs28577594
rs77231040
rs141214782
rs182059586
rs76065543
rs2056726
rs112394943
rs150150565
rs932002
rs6776756
rs139669835
rs2977608
rs12369950
rs117034449
rs11699829
rs78491606
rs28502153
rs61405042
rs73730598
rs11769630
rs6790988
rs7209057
rs76666449
rs11412296
rs73581419
rs10774624
rs76219171
rs3767952
rs45604339
rs6584579
rs79977579
rs6751209
rs9955360
rs4616688
rs6881568
rs4695407
All
rs28363070
rs188918174
rs6590343
rs2967355
rs66731853
rs145114957
rs11212631
rs9878436
rs202034370
rs17445108
rs80324517
rs11085072
rs55747751
rs9600019
rs80116508
rs12412214
rs1957937
rs10905255
rs871134
rs41304832
rs9940099
rs6659669
rs144204502
rs16978028
rs4498805
rs59409453
rs3093888
rs450962
rs1023767
rs2282764
rs11426156
rs4530278
rs6669563
rs77732866
rs11579626
rs4731541
rs113525195
rs2555104
rs8102497
rs117630647
rs34550383
rs10768683
rs117512405
rs6054257
rs139795227
rs137901416
rs17677991
rs2538745
rs7164950
rs1291143
rs56061761
rs5742915
rs9398196
rs12925933
rs11557154
rs10840270
rs142730696
rs762679
rs6536702
rs116863223
rs2763979
rs131797
rs611646
rs7790856
rs35446936
rs35640778
SNP
−0.04
−0.02
0.00
0.02
Effect size (b)
